# Evidence for Karyotype Polymorphism in the Free-Living Flatworm, *Macrostomum lignano*, a Model Organism for Evolutionary and Developmental Biology

**DOI:** 10.1371/journal.pone.0164915

**Published:** 2016-10-18

**Authors:** Kira S. Zadesenets, Dita B. Vizoso, Aline Schlatter, Irina D. Konopatskaia, Eugene Berezikov, Lukas Schärer, Nikolay B. Rubtsov

**Affiliations:** 1 Evolutionary Biology, Zoological Institute, University of Basel, Basel, Switzerland; 2 Institute of Cytology and Genetics SB RAS, Novosibirsk, Russian Federation; 3 European Research Institute for the Biology of Ageing, University of Groningen, University Medical Center Groningen, Groningen, The Netherlands; 4 Novosibirsk State University, Novosibirsk, Russian Federation; Universita degli Studi di Roma La Sapienza, ITALY

## Abstract

Over the past decade, the free-living flatworm *Macrostomum lignano* has been successfully used in many areas of biology, including embryology, stem cells, sexual selection, bioadhesion and aging. The increased use of this powerful laboratory model, including the establishment of genomic resources and tools, makes it essential to have a detailed description of the chromosome organization of this species, previously suggested to have a karyotype with 2n = 8 and one pair of large and three pairs of small metacentric chromosomes. We performed cytogenetic analyses for chromosomes of one commonly used inbred line of *M*. *lignano* (called DV1) and uncovered unexpected chromosome number variation in the form of aneuploidies of the largest chromosomes. These results prompted us to perform karyotypic studies in individual specimens of this and other lines of *M*. *lignano* reared under laboratory conditions, as well as in freshly field-collected specimens from different natural populations. Our analyses revealed a high frequency of aneuploids and in some cases other numerical and structural chromosome abnormalities in laboratory-reared lines of *M*. *lignano*, and some cases of aneuploidy were also found in freshly field-collected specimens. Moreover, karyological analyses were performed in specimens of three further species: *Macrostomum* sp. 8 (a close relative of *M*. *lignano*), *M*. *spirale* and *M*. *hystrix*. *Macrostomum* sp. 8 showed a karyotype that was similar to that of *M*. *lignano*, with tetrasomy for its largest chromosome being the most common karyotype, while the other two species showed a simpler karyotype that is more typical of the genus *Macrostomum*. These findings suggest that *M*. *lignano* and *Macrostomum* sp. 8 can be used as new models for studying processes of partial genome duplication in genome evolution.

## Introduction

Making progress in our understanding of biological processes often depends on the availability of suitable experimental model organisms. In theory, there are many species whose biology and natural history make them interesting models for specific research fields. Moreover, recent technical advances in our ability to perform genome sequencing (e.g. using next-generation sequencing), functional testing (e.g. using RNAi), and genome editing (e.g. using CRISPR/Cas9) are currently reshaping our conception of what makes a good model organism, as more and more former non-model organisms are making the transition to becoming officially accepted models (see e.g. http://www.nigms.nih.gov/Research/models). Thus, in addition to common features of model organisms, such as being amenable to be cultivated under laboratory conditions, having a small body-size and a short generation time, a stable genome organization would facilitate crucial genetic studies.

Over the past decade, the free-living flatworm *Macrostomum lignano* (Platyhelminthes, Rhabditophora) was introduced as a new model organism for research in evolutionary and developmental biology of the Lophotrochozoa [1–3]. This flatworm is small and transparent, with clearly defined organ systems, and it is easily cultured under laboratory conditions, making it very convenient for a diversity of research topics. As most species in the genus studied so far, *M*. *lignano* is an obligate outcrossing simultaneous hermaphrodite [4, 5], while other species, such as *M*. *hystrix*, though preferentially outcrossing are able to self-fertilize [6]. Both these features facilitate controlled crossing experiments. Furthermore, this flatworm has many additional beneficial traits, including a high regenerative potential provided by pluripotent stem cells, the so-called neoblasts [7, 8]. These cells permit efficient whole body regeneration after amputation of different body parts [9], and the regeneration process can be studied with functional assays [10, 11]. All these features make *M*. *lignano* an attractive and convenient model for many research fields of biology, from the study of embryonic development to aging processes. The establishment of laboratory lines and cultures of *M*. *lignano* was an important step in developing this new experimental model, as was the recently published genome of one inbred line, called DV1 [12]. To date, this inbred line, a transgenic line called HUB1 (established in the DV1 line), and a number of outbred cultures have been successfully maintained for years and used for many studies [1, 13–17].

As for any model organism, our understanding of *M*. *lignano* should include a detailed description of the karyotype obtained using different chromosome techniques. At the beginning of this study, we uncovered unexpected findings with respect to the *M*. *lignano* karyotype, which has previously been described as 2n = 8, with one pair of large and three pairs of small metacentric chromosomes [18]. These anomalies included aneuploidies (i.e. the presence of abnormal numbers of chromosomes in a cell) for the largest chromosomes in worms of the DV1 line. Later, unexpected inheritance patterns of the GFP marker in transgenic lines [17], as well as other unexpected patterns regarding the frequency distribution of different DNA motifs [12], prompted us to undertake more detailed karyotyping studies.

Here we present the results of cytogenetic and karyological analyses performed on (i) specimens from inbred lines and outbred cultures of *M*. *lignano* held in the laboratory, (ii) specimens freshly field-collected from natural populations, including a currently unnamed *Macrostomum* species (herein called *Macrostomum* sp. 8 and a close relative of *M*. *lignano*), and (iii) specimens from laboratory lines and cultures of two other, somewhat more distantly related species, *M*. *spirale* and *M*. *hystrix*. We document a high frequency of aneuploids in laboratory-reared lines of *M*. *lignano* and also found aneuploids in natural populations. Furthermore, we found that *Macrostomum* sp. 8 has a karyotype similar to that of *M*. *lignano*, with tetrasomy for its largest chromosome being the dominant karyotype, while the other two species have karyotypes that are more typical of the genus *Macrostomum*.

## Materials and Methods

### Study organisms

**Fig 1:**
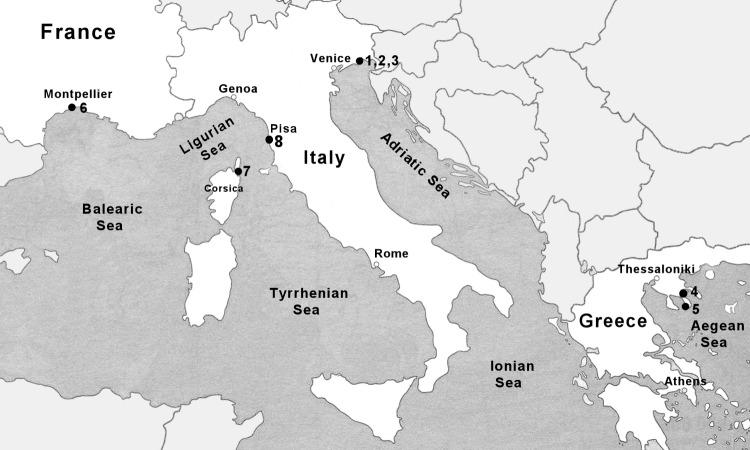
Map of the collection sites for the different *Macrostomum* species and specimens. The sites mentioned in Table 1 are identified by number: UV in Bibione (1), P1 in Lignano Sabbiadoro (2), and PS on Isola di Martignano (3), Vourvourou (4), Porto Koufo (5), Étang de Biguglia (6), Palavas-les-Flots (7), and San Rossore Regional Park (8).

We analyzed the karyotype of specimens from four related species of the genus *Macrostomum*: *M*. *lignano*, *M*. sp. 8, *M*. *spirale*, and *M*. *hystrix*, including seven different inbred lines and outbred cultures within *M*. *lignano*, our primary experimental model organism. In Table 1 we summarize the year and site of collection and the culture conditions for the different lines and cultures and in Fig 1 we show the collection sites of the different *Macrostomum* species and specimens. Sampling in the San Rossore Regional Park was performed under permit 3299/7-2-1 of the Tenuta di San Rossore, and all other sites did not include national parks or other protected areas of land or sea. Moreover, none of the field collections represent collections of endangered or protected species, samples were taking with minimal impact on the studied habitats, and the sampling did not include any vertebrates or cephalopods.

**Table 1:** *Macrostomum* species and specimens used in our study.

Culture	Year	Collection sites	Culture conditions	Reference
**(a) Inbred lines and outbred cultures of *M*. *lignano***				
Inbred lines				
DV1	2003	Bibione (site UV, N45.63405, E13.07626), Italy	32‰ f/2 in glass Petri dishes	[14]
HUB1		A transgenic line created from DV1	32‰ f/2 in glass Petri dishes	[15]
Outbred cultures				
LS1	2003	Bibione (site UV) and Isola di Martignano (site PS, N45.70383, E13.15793), Italy	32‰ f/2 in glass Petri dishes	[17]
LS2	2011	Bibione (close to site UV) and Lignano Sabbiadoro (close to site P1, N45.6918, E13.1312), Italy	32‰ f/2 in glass Petri dishes	this study
LS3	2013	Vourvourou (N40.2029, E23.7672) and Porto Koufo (N39.9595, E23.9278), Sithonia Peninsula, Greece	32‰ f/2 in glass Petri dishes	this study
IBK2	2012	Lignano Sabbiadoro (close to site P1), Italy collected by Peter Ladurner, Uni Innsbruck	32‰ f/2 in glass Petri dishes	this study
**(b) Freshly field-collected specimens of *M*. *lignano* and *Macrostomum* sp. 8**				
*M*. *lignano*	2014	Lignano Sabbiadoro (close to site P1)	32‰ ASW* in PS culture plates	this study
*Macrostomum* sp. 8	2014	Palavas-les-Flots (N43.4994, E3.8694)	32‰ ASW in PS culture plates	this study
**(c) Cultures of other *Macrostomum* species**				
*M*. *spirale* (outbred culture)	2004	Étang de Biguglia (N42.6591, E9.4504)	6‰ ASW in glass Petri dishes	this study
*M*. *hystrix* (inbred line SR1)	2010	San Rossore Regional Park (N43.6843, E10.2830), Pisa, Italy	6‰ ASW in glass Petri dishes	[6]

(a) inbred lines and outbred cultures of *Macrostomum lignano*

(b) freshly field-collected specimens of *M*. *lignano* and *Macrostomum* sp. 8, and

(c) cultures of other *Macrostomum* species (*M*. *spirale* and *M*. *hystrix*) used in our study, listing the year of establishment/collection, the site of collection, the laboratory culture conditions, and the reference if already published.

Sites UV, PS, and P1 are described in [1], although note that there were some errors in the reported coordinates for some collection sites.

ASW*–artificial sea water

All worms were kept in the laboratory at 20°C, and a light:dark cycle of 14:10 h, and fed with the diatom *Nitzschia curvilineata*, as previously described for *M*. *lignano* [1, 19].

The *M*. *lignano* inbred line DV1 has been widely used in different studies, including the recently published *M*. *lignano* genome project [12]. DV1 was created via full-sib and half-sib inbreeding for 24 generations, and has since been kept at small population sizes to maintain a high level of homozygosity [14]. DV1 was used to create a stable transgenic GFP(+) line, HUB1 [14, 15, 17]. Briefly, transgenesis was established by microinjecting the *Minos* transposon system into single-cell stage eggs. The reporter construct (PEfa::EGFP) contained the sequence of the *Minos* transposon, the promoter of the housekeeping gene elongation factor 1 alpha (*Efalpha*), and EGFP (encoding the enhanced GFP protein). After 48–72 h the injected embryos were screened for EGFP expression. Three injected eggs gave rise to three EGFP-transgenic lines and one of them became the HUB1 line [15].

In contrast to inbred lines, outbred *M*. *lignano* cultures are kept in a metapopulation structure in order to maximize the retention of genetic variability [17]. Freshly field-collected specimens (Table 1) of *M*. *lignano* and of a currently unnamed species of *Macrostomum* (here referred to as *Macrostomum* sp. 8, which recent molecular phylogenetic analyses have identified as a sibling species of *M*. *lignano*; T. Janssen and L. Schärer, unpublished data) were kept under laboratory conditions for up to two weeks after collection, before karyotype analyses.

More limited analyses were done on specimens from laboratory cultures of two more distantly related *Macrostomum* species (Table 1). According to the current molecular phylogeny of the genus *Macrostomum*, both *M*. *spirale* and *M*. *hystrix* fall into the same subclade of the genus (clade 2 in [13]; see also there for notes on the taxonomic status of these species names), with *M*. *hystrix* being considerably closer to *M*. *lignano* than *M*. *spirale*.

### Metaphase chromosome preparation

Chromosome slides were prepared using two basic techniques, modified after a previously described protocol [18], an important aspect of which is the induction of regeneration and subsequent formation of a regeneration blastema in order to increase the number of dividing cells [20].

The first technique, single-worm karyotyping, was used to obtain metaphase chromosome preparations from single worms in order to accurately describe the karyotypes of individual animals. We only analyzed specimens for which we could obtain at least 10 metaphase plates, thus permitting us to evaluate whether there were any cases of within-individual mosaicism and hence increasing the certainty of the presented karyotypes. To induce regeneration, adult animals were cut transversely at the level of the ovaries and their anterior parts were allowed to regenerate for 12–18 h. The regenerating worm fragments were then treated with 0.2% (w/v) colchicine (Carl Roth, Germany) solution in f/2 medium for 1 h at room temperature (RT) to arrest mitosis in dividing cells at the metaphase stage. The worm fragments were then treated with hypotonic 0.2% (w/v) KCl solution, to induce cell swelling (1–1.5 h at RT). Each worm fragment was placed onto a dry clean slide in a mix of 3:3:4 parts of glacial acetic acid: ethanol: distilled water and then macerated into small pieces with glass needles made of pulled glass Pasteur pipettes to distribute the cells on the slide. Next, 20 01μl of a mix of 1:1 parts of glacial acetic acid: ethanol was dropped onto the material on the slide, immediately followed by 20 μl of pure glacial acetic acid, and then the slide was placed horizontally in a humidity chamber for 2–3 min. Finally, the slide with the fixed material was dried for 5–10 min at 60°C.

The second technique, cell-suspension karyotyping, was used to obtain large numbers of metaphase spreads and therefore required many animals. Such preparations were used for metaphase chromosome microdissection or FISH approaches, according to a slightly modified technique developed for the preparation of opisthorchid mitotic and meiotic chromosomes (the ‘cell suspension’ method) [21]. In the present study, adult worms, cut one day before as above, were treated with distilled water (instead of hypotonic 0.56% KCl solution used by [21]) for 20–30 min at RT. For such metaphase chromosome preparations usually 150–200 worms were used.

To investigate the possibility that chromosome number variation could have arisen due to unusual mitotic divisions within the regeneration blastema, we also prepared chromosome slides without the induction of regeneration. While this greatly decreased the number of metaphase plates we found per individual, each individual still displayed chromosome plates with a consistent chromosome number.

### Metaphase chromosome staining and microscopy analysis

For karyotyping, the chromosome slides were stained using fluorescent DNA dyes, either 4',6-diamidino-2-phenylindole (DAPI) and / or chromomycin A3 (CMA), according to standard protocols [22]. Note that CMA and DAPI are GC- and AT-specific, and thus lead to stronger staining of GC- and AT-rich regions, respectively [23]. For microdissection (see below), the chromosome slides were stained with 0.1% Giemsa stain (Sigma) for 3–5 min at RT.

Images of fluorescently stained metaphase chromosomes were captured using either (i) a CCD-camera installed on a Axioplan 2 compound microscope (Carl Zeiss, Germany) equipped with filtercubes #49, #10, and #15 (ZEISS, Germany) using AxioVision (Carl Zeiss, Germany) or ISIS4 (METASystems GmbH, Germany) at the Multiple-access Center for Microscopy of Biological Subjects (Institute of Cytology and Genetics, Novosibirsk, Russia); or (ii) a Leica DFC 360 FX camera (Leica Microsystems CMS GmbH) installed on a Leica DM 2500 compound microscope with filtercube B/G/R (Leica Microsystems) using LAS V4.1 (Leica Microsystems) at the Zoological Institute, University of Basel, Switzerland.

### Morphometric analysis

Morphometric measurements of the length of metaphase chromosomes were carried out on captured images using MicroMeasure 3.3 [24]. Morphometric measurements, according to standard nomenclature [25], included absolute length of individual chromosomes (AL), relative length (RL = AL x 100% / half the length of all chromosomes in a metaphase spread), lengths of the short and long arms (S and L, respectively), arm length ratio, (R = L/S), and centromeric index (CI = S/(L+S)). Note that the chromosomes of all species studied here were classified by decreasing size in pairs according to the data of the morphometric analysis. Thus the way in which we classified the chromosomes may to some degree have led to an overestimation of the size differences between them.

### DNA probes and fluorescence *in situ* hybridization

A number of different DNA probes were generated for fluorescence *in situ* hybridization (FISH) analyses in the main study species, *M*. *lignano* and its close relative *Macrostomum* sp. 8. A telomere DNA probe was generated by PCR in the absence of DNA template using primers (TTAGGG)_5_ and (CCCTAA)_5_ following the standard protocol [26]. DNA labelling was performed with TAMRA-dUTP (Genetyx, Novosibirsk) in additional PCR cycles [26]. For FISH localization of the ribosomal DNA cluster, the primers WormA (5-GCGAATGGCTCATTAAATCAG-3) and WormB (5-CTTGTTACGACTTTTACTTCC-3) were used to amplify a 1177 bp fragment corresponding to a part of the 28S rDNA gene of *M*. *lignano* [13]. The generated DNA probe was labeled in additional PCR cycles with specific primers in the presence of Flu-dUTP (Genetyx, Novosibirsk).

Chromosome microdissection and amplification of DNA isolated from these chromosomes by degenerate oligonucleotide primed polymerase chain reaction (DOP-PCR) were carried out as described previously [27]. Microdissected DNA probes, *Mli1* and *Mlism*, were generated from eight large and all small chromosomes from eight metaphase spreads of *M*. *lignano*, respectively. The obtained PCR products were labeled with Flu- or TAMRA-dUTP (Genetyx, Novosibirsk) in additional PCR cycles.

FISH with DNA probes on metaphase chromosomes of *M*. *lignano* was performed as described earlier [21] with salmon sperm DNA as a DNA carrier, without Cot1 DNA (DNA enriched for repetitive DNA sequences) to suppress of repetitive DNA hybridization. Chromosomes were counterstained with DAPI dissolved in Vectashield antifade solution (Vector Laboratories, USA).

### Karyotype frequencies and karyotype inheritance

The observed frequencies of the three main karyotypes in the populations of *M*. *lignano* (i.e. 2n = 8, 2n = 9, and 2n = 10, for details see results) allow us to evaluate the hypothesis that additional large chromosomes follow a simple Mendelian inheritance (note that the rare 'abnormal karyotypes' were excluded for the following considerations). If we assume fair meiosis then 2n = 8, 2n = 9, and 2n = 10 individuals are expected to produce n = 4 and n = 5 gametes in the ratios of 100% vs. 0%, 50% vs. 50%, and 0% vs. 100%, respectively. We can thus generate an expectation for the frequency of n = 4 (p) and n = 5 (q) gametes in the population and the resulting frequencies of 2n = 8 (p^2^), 2n = 9 (2pq), and 2n = 10 (q^2^) offspring. The observed vs. expected frequencies can then be tested with a *Χ*^*2*^-test for independent assortment. A deviation from the expected frequencies might either indicate that gametes are not being generated at the expected frequencies (e.g. due to unfair meiosis), do not fuse at random (e.g. due to haploid selection or assortative mating), or that there is selection among the resulting zygotes (e.g. selection against certain karyotype combinations). Such analyses were done separately for the DV1 and the HUB1 line, followed by a Fisher’s combined probability test to evaluate the overall evidence.

To more directly understand the inheritance of additional large chromosomes to the offspring and assess the fertility of aneuploid worms, we performed crossing experiments between DV1 worms whose karyotypes were determined before crossing. Briefly, worms were cut in half between the ovaries and testes, yielding ‘head fragments’ and ‘tail fragments’. The head fragments were isolated in wells of 24-well cell culture plates (Eppendorf, Germany) and permitted to regenerate their whole body (which at that cutting level takes about two weeks) [9], while the tail fragments were used for chromosome preparation (as outlined above). This allowed us to determine the karyotypes of worms while keeping them alive and made sure that the worms could be considered 'virgins' (as both male and female genitalia had to regenerate). We tested all cross combinations involving one 2n = 9 individual as one parent and a 2n = 8, 2n = 9, or 2n = 10 individual as the other parent (at least 5 pairs per cross combination). Pairs were transferred every 7 days to new wells with fresh algae, and the old wells were screened for hatchlings every 3 days. Hatchlings were isolated in new wells until they reached sexual maturity, after which they were karyotyped (at least 25 specimens per cross combination) using whole worms for chromosome preparation. We used a *Χ*^*2*^-test to compare the observed number of resulting offspring with the different karyotypes to the expected frequencies given the karyotypes (and hence expected gamete frequencies) of their known parental individuals.

### Statistical analysis

All statistical analyses were carried out using commercially available software (STATISTICA version 13 by StatSoft Inc., USA).

## Results

### Morphometric analysis

**Fig 2:**
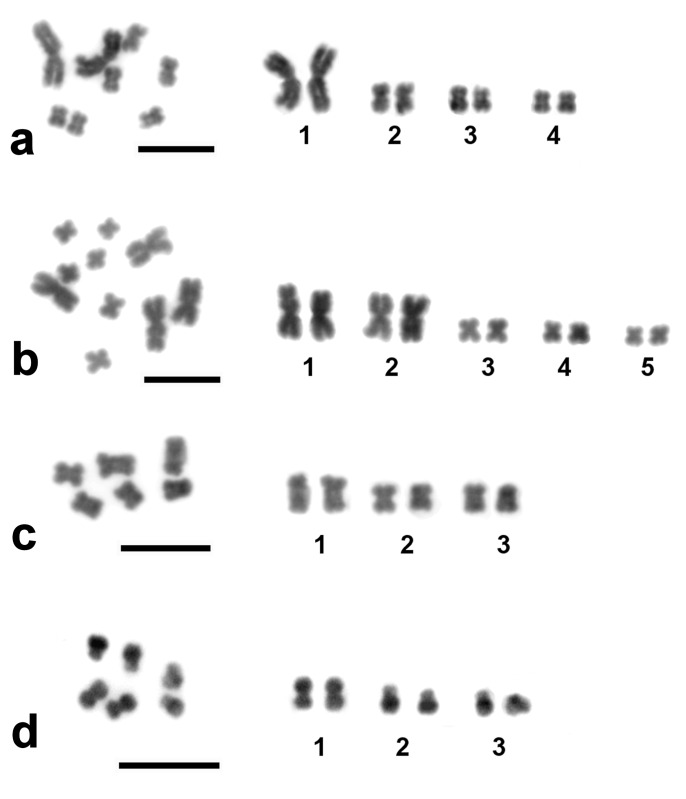
Karyotype variation among *Macrostomum* species. (a) the 'normal' chromosome set of *Macrostomum lignano*, 2n = 8, (b) the 'normal' chromosome set of *Macrostomum* sp. 8, 2n = 10, and the invariant chromosome sets of (c) *M*. *spirale*, 2n = 6, and (d) *M*. *hystrix*, 2n = 6. DAPI-staining (inverted image). Scale bar 10 μm.

For the morphometric analyses 50 metaphase plates derived from 30 specimens of *M*. *lignano* and 35 metaphase plates derived from 18 specimens of *Macrostomum* sp. 8 were measured (Table 2). All specimens used for morphometry were freshly collected from natural populations and we only used worms that showed the 'normal' chromosome numbers (i.e. 2n = 8 for *M*. *lignano* and 2n = 10 for *Macrostomum* sp. 8, see next section for details about karyotype variation). For all species, we organized the chromosome pairs by decreasing size, with the largest being chromosome pair 1 (Fig 2).

**Table 2:** Morphometric analysis of *Macrostomum* karyotypes.

	AL (μm)	RL (%)	L (μm)	S (μm)	R	CI
**(a) *Macrostomum lignano***						
1	5.27 ± 0.81	41.24 ± 1.5	2.81 ± 0.46	2.46 ± 0.39	1.15 ± 0.13	0.47 ± 0.03 (m)
2	2.74 ± 0.27	21.54 ± 0.63	1.47 ± 0.16	1.26 ± 0.16	1.18 ± 0.16	0.46 ± 0.03 (m)
3	2.49 ± 0.25	19.59 ± 0.56	1.34 ± 0.15	1.15 ± 0.13	1.17 ± 0.11	0.46 ± 0.02 (m)
4	2.24 ± 0.29	17.62 ± 0.01	1.19 ± 0.14	1.06 ± 0.19	1.13 ± 0.12	0.47 ± 0.07 (m)
**(b) *Macrostomum* sp. 8**						
1	6.34 ± 0.35	32.28 ± 0.61%	3.29 ± 0.13	3.05 ± 0.21	1.06 ± 0.06	0.43 ± 0.06 (m)
2	5.89 ± 0.39	31.62 ± 1.08%	2.89 ± 0.19	2.86 ± 0.21	1.07 ± 0.09	0.47 ± 0.01 (m)
3	2.98 ± 0.02	14.16 ± 0.01%	1.55 ± 0.01	1.43 ± 0.03	1.11 ± 0.01	0.47 ± 0.01 (m)
4	2.83 ± 0.09	13.54 ± 0.25%	1.59 ± 0.09	1.24 ± 0.19	1.37 ± 0.19	0.42 ± 0.02 (m)
5	2.36 ± 0.02	11.76 ± 0.86%	1.23 ± 0.06	1.13 ± 0.04	1.08 ± 0.02	0.45 ± 0.02 (m)
**(c) *Macrostomum spirale***						
1	3.75 ± 0.47	36.72 ± 1.35	2.06 ± 0.35	1.67 ± 0.20	1.22 ± 0.18	0.45 ± 0.03 (m)
2	3.37 ± 0.34	33.11 ± 0.91	1.87 ± 0.26	1.57 ± 0.28	1.22 ± 0.12	0.45 ± 0.02 (m)
3	3.07 ± 0.28	30.16 ± 1.13	1.67 ± 0.17	1.45 ± 0.27	1.20 ± 0.12	0.45 ± 0.02 (m)
**(d) *Macrostomum hystrix***						
1	3.76 ± 0.54	38.07 ± 1.34	2.09 ± 0.37	1.78 ± 0.32	1.26 ± 0.37	0.46 ± 0.03 (m)
2	3.31 ± 0.48	32.67 ± 1.19	2.1 ± 0.35	1.27 ± 0.27	1.85 ± 0.34	0.37 ± 0.06 (sm)
3	2.96 ± 0.45	29.26 ± 1.08	1.94 ± 0.36	1.04 ± 0.18	1.90 ± 0.30	0.35 ± 0.04 (sm)

(a) *Macrostomum lignano* based on 'normal' 2n = 8 metaphase plates (N = 50)

(b) its close relative *Macrostomum* sp. 8 based on 'normal' 2n = 10 metaphase plates (N = 35), and two more distantly related species, namely

(c) *M*. *spirale* 2n = 6 (N = 20) and

(d) *M*. *hystrix* 2n = 6 (N = 20).

The reported values represent means±1SD and include the absolute length of each chromosome (AL), the relative length of each chromosome, the length of the long arm (L), the short arm (S) and the arm ratio (R = L/S); and the centromeric index [CI = S/(L+S)] (m, sm stand for metacentric and submetacentric, correspondingly).

The morphometric analysis showed that the 'normal' karyotype of *M*. *lignano* consists of one chromosome pair being much larger than the other three, and all of them being metacentrics (Fig 2A). The largest chromosome is more than twice the length of the smallest, while the other two chromosomes are just slightly larger than the latter (Table 2). Both the chromosome number and their relative lengths are in fairly good agreement with those reported earlier [18] for an outbred culture collected in the same general area [1].

Despite being a close relative of *M*. *lignano* the karyotype of *Macrostomum* sp. 8—described for the first time in this study—was clearly distinct. Its 'normal' karyotype is 2n = 10, containing two pairs of large chromosomes and three pairs of small chromosomes, all of them being metacentrics (Fig 2B, Table 2). The large chromosomes of *Macrostomum* sp. 8 are similar in size, while one of the small chromosomes is again smaller than the other two (Table 2).

For the morphometric analyses in the more distantly related *Macrostomum* species, we measured 20 plates from 15 specimens of *M*. *spirale* and 20 plates from 10 specimens of *M*. *hystrix* (Table 2). Both species have a karyotype of 2n = 6, consisting of chromosomes that gradually decrease in size (Fig 2C and 2D; Table 2). The karyotype of *M*. *spirale* consists of three pairs of metacentric chromosomes, while the karyotype of *M*. *hystrix* consists of one pair of metacentric and two pairs of submetacentric chromosomes. In both *M*. *spirale* and *M*. *hystrix* these chromosomes are similar in size to the small chromosomes of *M*. *lignano* and *Macrostomum* sp. 8 (see Discussion).

As pointed out already in the Materials and Methods section, the way in which we classified the chromosomes (according to their size) may to some degree have led to an overestimation of the size differences between them. The absolute length of the chromosomes and chromosome arms in metaphase spreads depended on the stage of mitosis, the position of the adjacent chromosomes, and their position in the metaphase spread. This means that it may not always have been possible to distinguish among chromosomes having similar parameters (size, centromere location), namely, among the three small metacentrics in *M*. *lignano* and *Macrostomum* sp. 8, and also among the two large metacentrics in *Macrostomum* sp. 8.

### Karyotype variation of inbred lines and outbred cultures of *Macrostomum lignano*

Based on previously reported results [18] on the karyotype of *M*. *lignano* we would have expected all worms belonging to the inbred DV1 line to have a karyotype of 2n = 8, with two large and six small metacentric chromosomes (what we refer to as the 'normal' karyotype of *M*. *lignano*). However, among 100 scored metaphase plates obtained by applying the cell-suspension karyotyping technique >100 worms of the inbred DV1 line we found many karyotypes with either 2n = 9 (49%) or 2n = 10 (31%), with the expected 2n = 8 karyotype being the least frequent (20%). To determine if this aneuploidy could have resulted from somatic mosaicism within some worms, from karyotype diversity among individual worms, or from a combination of the two, a total of six laboratory lines and cultures of *M*. *lignano* (Table 3) and also freshly field-collected specimens of *M*. *lignano* and *Macrostomum* sp. 8 (see next section, Table 3) were subsequently analyzed with the single-worm karyotyping technique. According to the origin and imposed breeding system the established laboratory populations of *M*. *lignano* are divided into two groups, namely inbred lines (DV1 and HUB1) and outbred cultures (LS1, LS2, LS3, and IBK2; discussed further below).

**Table 3:** Chromosome number variation in individually karyotyped specimens.

Line/culture/ field collection	n	'normal' karyotype n (%)	plus one large metacentric n (%)	plus two large metacentrics n (%)	'abnormal' karyotypes n (%)	*X*^2^	*DF*	*P*
**(a) Inbred lines and outbred cultures of *M*. *lignano***								
Inbred lines								
DV1	134	16 (11.9%)	77 (57.5%)	36 (26.9%)	5 (3.7%)			
(observed)*	129	16 (12.4%)	77 (59.7%)	36 (27.9%)	-	6.43	1	**0.011**
(expected)*		(17.8%)	(48.8%)	(33.4%)	-			
HUB1	137	18 (13.1%)	72 (52.6%)	42 (30.7%)	5 (3.6%)			
(observed)*	132	18 (13.6%)	72 (54.5%)	42 (31.4%)	-	2.17	1	0.141
(expected)*		(16.7%)	(48.3%)	(34.9%)	-			
Outbred cultures								
LS1	285	276 (96.8%)	5 (1.8%)	-	4 (1.4%)			
LS2	61	61 (100%)	-	-	-			
LS3	50	23 (46%)	6 (12%)	17 (34%)	4 (8%)			
IBK2	50	44 (88%)	1 (2%)	-	5 (10%)			
**(b) Freshly field-collected specimens**								
*M*. *lignano*	122	120 (98.4%)	1 (0.8%)		1 (0.8%)			
*Macrostomum* sp. 8	22	18 (81.8%)	2 (9.1%)	-	2 (9.1%)			
**(c) Cultures of other *Macrostomum* species**								
*M*. *spirale* (2n = 6)	97	91 (100%)	-	-	-			
*M*. *hystrix* (2n = 6)	10	10 (100%)	-	-	-			

(a) inbred lines and outbred cultures of *M*. *lignano*

(b) freshly field-collected specimens of *M*. *lignano* and *Macrostomum* sp. 8, and

(c) cultures of other *Macrostomum* species (*M*. *spirale* and *M*. *hystrix*).

The 'normal' karyotype is 2n = 8 in *M*. *lignano* (two large and six small metacentrics), 2n = 10 in *Macrostomum* sp. 8 (four large and six small metacentrics), 2n = 6 in *M*. *spirale* (six metacentrics), and 2n = 6 in *M*. *hystrix* (two metacentrics and four submetacentrics). For both the DV1 and HUB1 lines we further provide a test for deviations from the expected karyotype frequencies (for rationale of test see main text).

Note that the asterisks (*) indicate that we have excluded the 'abnormal' karyotypes for these calculations.

Based on the observed karyotype frequencies among the analyzed individuals the expected frequencies for n = 4 and n = 5 gametes are p = 0.422 and q = 0.578 for DV1 and p = 0.409 and q = 0.591 for HUB1, respectively, permitting to calculate the expected karyotype frequencies (i.e. p^2^, 2pq, and q^2^ for the 2n = 8, 2n = 9, and 2n = 10 karyotypes, respectively).

**Fig 3:**
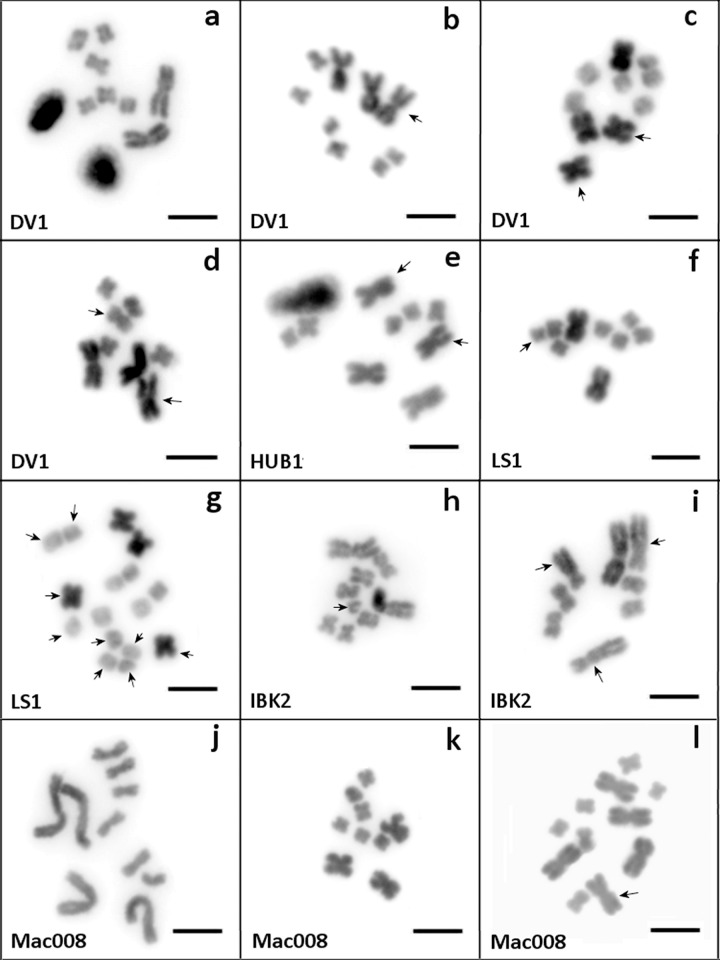
Karyotype diversity among individually karyotyped specimens of *Macrostomum lignano* and *Macrostomum* sp. 8, based on ≥10 chromosome plates per specimen. Karyotype diversity of *Macrostomum lignano* (a-i). (a) 'normal' 2n = 8 (two large and six small metacentrics); (b) 2n = 9 (three large and six small metacentrics); (c) 2n = 10 (four large and six small metacentrics); (d) 'abnormal' 2n = 8 (three large and five small metacentrics); (e) 'abnormal' 2n = 9 (four large and five small metacentrics); (f) 'abnormal' 2n = 9 (two large and seven small metacentrics); (g) 'abnormal' 2n = 16 (four large and twelve small metacentrics); (h) 'abnormal' 2n = 9 (two large, six small metacentrics and one extra small chromosome); (i) 'abnormal' 2n = 8 (one large metacentric, three medium-sized submetacentrics, and four small metacentrics). Karyotype diversity of *Macrostomum* sp. 8 (j-l). (j) 'normal' 2n = 10 (four large and six small metacentrics); (k) 2n = 9 (three large and six small metacentrics); (l) 2n = 11 (five large and six small metacentrics). Chromosome rearrangements are marked with arrows. DAPI-staining (inverted image). Scale bar 10 μm.

Both studied inbred lines, DV1 and HUB1 (see Table 1 for details), were characterized by a comparably high frequency of aneuploid worms (Table 3) and the complete absence of any somatic mosaicism, with the most abundant karyotype containing one additional large chromosome (total chromosome number 2n = 9; Fig 3B), the second most abundant karyotype containing two additional large chromosomes (total chromosome number 2n = 10; Fig 3C), and the 'normal' 2n = 8 karyotype (Fig 3A) being relatively rare. Furthermore, in both lines we found a few specimens with other karyotype variants (further called 'abnormal' karyotypes) (Table 3). These abnormalities included additional rearranged chromosomes of unknown origin, including metacentrics and submetacentrics of different sizes, and simultaneous aneuploidies of large and small chromosomes (e.g. Fig 3D and 3E). It should be noted that no evident morphological or behavioral abnormalities were observed in the specimens carrying these abnormal karyotypes (see also below). The fact that the frequencies of the different karyotypes are so similar between the DV1 line and transgenic HUB1 line is likely due to the latter having been derived from the former.

The karyotype frequencies of DV1 specimens deviated significantly from those expected under independent assortment of n = 4 and n = 5 gametes, with the 2n = 8 karyotype being rarer than expected (Table 3). A similar, but non-significant trend was evident among the HUB1 line individuals (Table 3), together yielding a highly significant Fisher's combined probability of *P* = 0.0016 [*Χ*^*2*^ = -2*(ln(0.011)+ln(0.141)) = 12.94 with *DF* = 2].

The crossing experiment between aneuploid worms with 2n = 9 and worms with all three main karyotypes (i.e. 2n = 8, 2n = 9, and 2n = 10) confirmed that aneuploid worms are fertile, as they produced viable progeny in all three combinations. Moreover, all of these progeny carried karyotypes that were expected if the additional large chromosomes are normally inherited (Table 4). This suggests that these aneuploid 2n = 9 worms do indeed produce viable n = 4 and n = 5 gametes. However, consistent with the population analysis shown above, we found evidence that fewer than expected 2n = 8 offspring resulted from 2n = 9 x 2n = 9 crosses, while the other two crosses did not deviate significantly from the expected frequencies (Table 4). We explore possible reasons for the lower than expected frequency of 2n = 8 individuals in the Discussion.

**Table 4:** Inheritance patterns of additional large chromosomes from one aneuploid 2n = 9 parent crossed in all combinations with another parent with one of the three main karyotypes.

Cross combination	n/n*	2n = 8 n (%)	2n = 9 n (%)	2n = 10 n (%)	Pearson *X*^2^	*DF*	*P*
2n = 9 x 2n = 8	25/5	15 (60%)	10 (40%)	-	1.00	1	0.3173
(expected)		(50%)	(50%)	-			
2n = 9 x 2n = 9	87/7	8 (9.2%)	54 (62.1%)	25 (28.7%)	**11.71**	**2**	**0.0029**
(expected)		(25%)	(50%)	(25%)			
2n = 9 x 2n = 10	25/5	-	16 (61.5%)	10 (38.5%)	1.38	1	0.2393
(expected)		-	(50%)	(50%)			

Also indicated are tests for a deviation for the expected karyotypes under Mendelian inheritance of the additional large chromosomes.

n/n*—number of karyotyped progeny/ number of crosses per each cross type

Among the outbred cultures, the proportion of specimens with different karyotypes deviated substantially from those in the inbred lines, with the 'normal' 2n = 8 karyotype being the most abundant in all cultures (Table 3). While no karyotypes other than the 'normal' 2n = 8 karyotype were revealed within the sampled specimens of the LS2 culture, other karyotypes, including some 'abnormal' karyotypes, were detected in the other outbred cultures (Table 3). In the LS1 culture, 5 of 285 worms had a 2n = 9 karyotype (three large and six small metacentrics) and 4 worms showed 'abnormal' karyotypes, namely an 'abnormal' 2n = 9 (two large and seven small metacentrics), 2n = 12 (three large and nine small metacentrics), and 2n = 16 (four large and twelve small metacentrics). Although the most frequent karyotype in the IBK2 culture was the 'normal' 2n = 8 karyotype, it actually showed the highest percentage of 'abnormal' karyotypes (Table 3), with different variants of rearranged chromosomes, including additional submetacentrics. For example, Fig 3I shows a metaphase spread containing 8 chromosomes, of which one being a large and unpaired metacentric, two making up a pair of medium-sized submetacentrics, one being medium-sized and unpaired submetacentric, and the remaining two making up a pair of small metacentrics. Finally, the LS3 culture showed the lowest proportion of 'normal' 2n = 8 karyotypes amongst the outbred cultures, and was the only outbred culture that showed any 2n = 10 karyotypes (with four large and six small metacentrics), which occurred in about one third of the worms (Table 3). In summary, our results suggest that our laboratory cultures, in addition to sometimes carrying aneuploidy, may also exhibit some level of structural chromosome rearrangements.

### Karyotype variation in freshly field-collected *Macrostomum lignano* and *Macrostomum* sp. 8

In addition to the laboratory lines and cultures of *M*. *lignano*, we also analyzed specimens of *M*. *lignano* and its sibling species *Macrostomum* sp. 8 freshly collected from natural populations (Tables 1 and 3). In *M*. *lignano*, the great majority of specimens carried the 'normal' 2n = 8 karyotype, but we also found two 2n = 9 worms (one with the commonly observed karyotype with three large and six small metacentrics, and the other with an 'abnormal' karyotype with two large and seven small metacentrics).

In contrast, the analyses of the karyotype of *Macrostomum* sp. 8 revealed that its chromosome number varied from 2n = 9 to 2n = 11 (Table 3), with the most abundant karyotype being 2n = 10 (four large and six small metacentrics; Fig 3J). This karyotype variant, which based on its high frequency could be considered the 'normal' karyotype, was accompanied by rarer karyotypes, including 2n = 9 (three large and six small metacentrics; Fig 3K, listed under 'abnormal' karyotypes in Table 3) and 2n = 11 (five large and six small metacentrics; Fig 3L, listed under 'plus one large metacentric' in Table 3).

### Karyotypes of other *Macrostomum* species

All of the karyotyped specimens of our laboratory cultured *M*. *spirale* (n = 97) and *M*. *hystrix* (n = 10) showed a 2n = 6 karyotype, which is the most frequently observed karyotype in the genus *Macrostomum* [18], and no karyotype variants were revealed among these species. However, given the comparably low numbers of karyotyped individuals, this finding does not permit us to make a strong claim that karyotype variation is completely absent in these species.

### Chromosome staining of *Macrostomum lignano* and *Macrostomum* sp. 8 using DAPI and CMA

**Fig 4:**
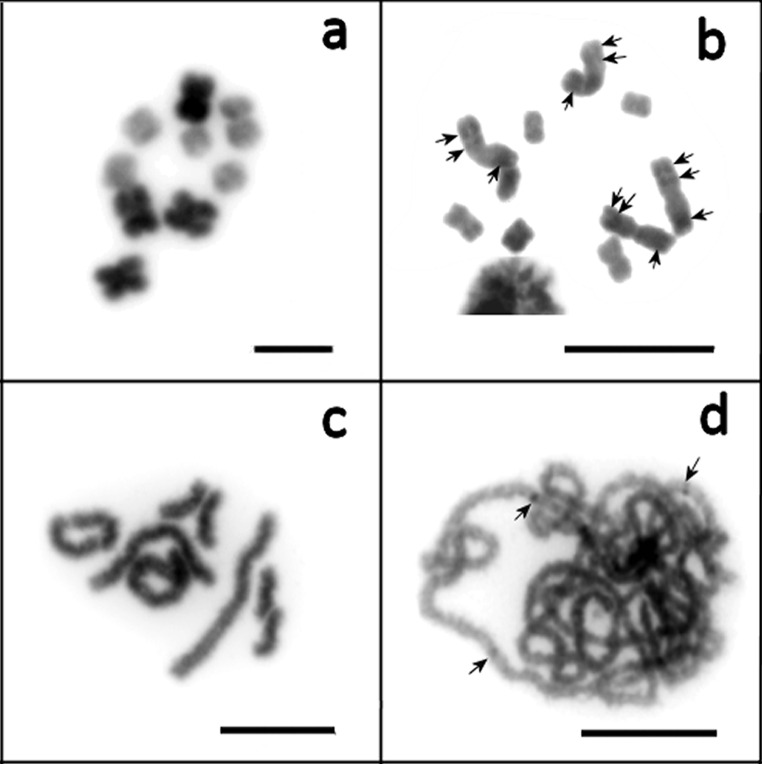
Chromosomes of *Macrostomum lignano* at different condensation levels, stained with DAPI (inverted image). (a-c) mitotic metaphase chromosomes; (d) pachytene chromosomes. AT-positive bands are marked with arrows. Scale bar 10 μm.

**Fig 5:**
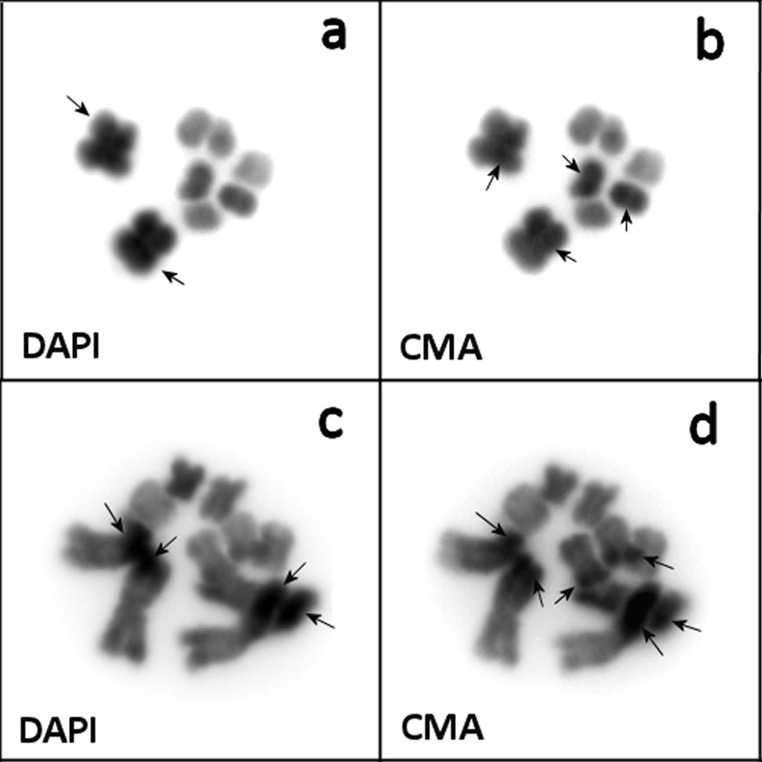
**Chromosomes of *Macrostomum lignano* (a, b) and *Macrostomum* sp. 8 (c, d) stained with both DAPI and CMA (inverted images).** Intensively stained chromosomal material is marked with arrows.

On metaphase plates containing highly-condensed chromosomes, the large chromosomes showed a more intense DAPI-signal than the small chromosomes (Figs 4A and 5A). This difference was less obvious for less-strongly condensed chromosomes, in which DAPI-positive (and likely AT-rich) regions were present in both arms of the large chromosomes, namely two in the short arm and one in the long arm (Fig 4B). Moreover, it should be noted that the DAPI-signal was more intense on one pair of the small chromosomes compared to the other small chromosomes (Figs 4B and 5A). On pachytene chromosomes, narrow AT-rich centromeric regions suggest that there are small clusters of repeats that are neighboring the centromeres (Fig 4D).

Given that CMA-staining of metaphase chromosomes of *M*. *lignano* was also more intense on the large chromosomes and one pair of the small chromosomes (Fig 5B), this suggests that the level of condensation of these chromosomes was higher than that in the remaining small chromosomes.

The images of the chromosomes of *Macrostomum* sp. 8 with combined DAPI- and CMA-staining showed results that were slightly different from those of *M*. *lignano*. One pair of the small chromosomes and the long arms of the large chromosomes appeared to be stained more intensely with CMA. This suggests that the terminal part of the long arm of one pair of small chromosomes contains a GC-rich region and may be enriched with genes. Moreover, intense staining with both dyes revealed that there is highly condensed chromosomal material in the long arm of the large chromosomes.

### DNA probes and fluorescence *in situ* hybridization

**Fig 6:**
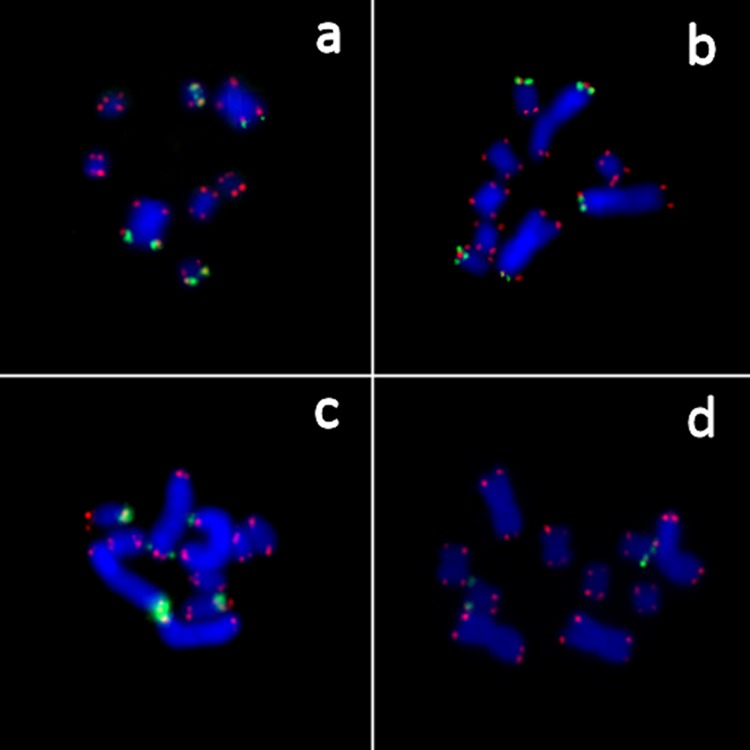
**Localization of clusters of 28S rDNA (green) and telomeric (red) repeats in chromosomes of *M*. *lignano* (a-c) and *Macrostomum* sp. 8 (d), using fluorescence *in situ* hybridization (FISH).** The chromosomes were stained with DAPI (blue colour).

Clusters of ribosomal (28S rDNA) and telomeric repeats could be successfully localized on metaphase chromosomes of *M*. *lignano* and *Macrostomum* sp. 8 using FISH with corresponding DNA probes (Fig 6). All clusters of telomeric repeats were found to be localized at the ends of chromosomes, and no telomeric repeats in interstitial sites were observed.

Clusters of 28S rDNA were revealed at the end of one arm of only one pair of the small chromosomes in both, *M*. *lignano* and *Macrostomum* sp. 8. In the checked specimens, these clusters were usually rather small in size, but some larger clusters were also found. Moreover, in *M*. *lignano* additional 28S rDNA clusters were found at the end of the short arm of the large chromosomes. These varied in size from very small to rather large (Fig 6A, 6B and 6C). Conversely, no 28S rDNA clusters were found at the end of the large chromosomes in any specimens of *Macrostomum* sp. 8 (Fig 6D). However, it is necessary to point out that we cannot exclude the possibility that a few copies of 28S rDNA genes may be present in other locations given the limited level of sensitivity of the FISH technique used in this study.

**Fig 7:**
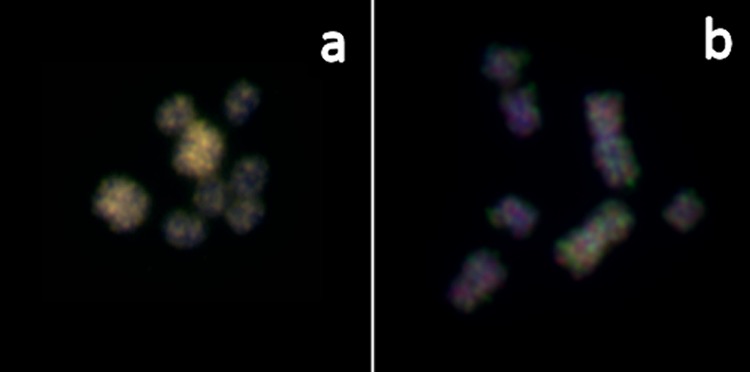
**Fluorescence *in situ* hybridization (FISH) using microdissected DNA probes obtained from the large chromosome (*Mli1*—green) and the small chromosomes (*Mlism*–red) on either highly condensed (a) and less-highly condensed (b) chromosomes of *M*. *lignano*.** The chromosomes were stained with DAPI (blue colour).

Two-color FISH with the obtained microdissected *Mli1* and *Mlism* DNA probes was performed without suppression of repetitive DNA sequences on metaphase chromosomes of *M*. *lignano*. As a result, a strong background signal was observed on all chromosomes. In highly condensed chromosomes of metaphase spreads more intense FISH signals of both DNA probes were revealed on large chromosomes, whereas on less condensed chromosomes the intensity of signals on large and small chromosomes was similar (Fig 7). This difference could be explained by a higher DNA concentration in condensed chromosome regions. It should be noted that FISH with microdissected DNA probes did not reveal regions of intense signal on low condensed chromosomes of *M*. *lignano*, which would be typical for large clusters of repeats.

## Discussion

Currently, there is relatively limited data on karyotypes of species in the genus *Macrostomum*, but according to the performed studies, summarized in [18], the dominant pattern for the genus is a karyotype consisting of 2n = 6, with three pairs of similar sized (sub)metacentrics, while a notable exception to this pattern is *M*. *hustedi* of Jones 1944, a species which has 2n = 12. When we view the karyotype data assembled by Egger and Ishida [18] together with the karyotype data presented here in the context of the current *Macrostomum* molecular phylogeny [13], it appears very probable that the karyotype of *M*. *lignano* (and *Macrostomum* sp. 8) has evolved from a 2n = 6 karyotype. Specifically, clade 2 (i.e. the clade containing *M*. *lignano* and *Macrostomum* sp. 8) also contains *M*. *tuba* and *M*. *finlandense* (which Egger and Ishida list as 2n = 6) as well as *M*. *spirale* and *M*. *hystrix* (which we here find to be 2n = 6). In contrast, although not yet phylogenetically placed, *M*. *hustedi* shows an anatomical organization that is characteristic of species in clade 1 [13]. Under the assumption that 2n = 6 is the ancestral karyotype, at least in clade 2, it is interesting to explore how the formation of the *M*. *lignano* and *Macrostomum* sp. 8 karyotypes might have taken place from that ancestral karyotype.

According to the morphometric data presented in this study, the karyotypes in *M*. *lignano* and *Macrostomum* sp. 8 show differently-sized chromosomes, while in both *M*. *spirale* and *M*. *hystrix* the chromosomes all have approximately similar sizes. It therefore seems plausible that the large chromosomes in *M*. *lignano* and *Macrostomum* sp. 8 represent a fusion product of some (or much) of the chromosomal material of a basal ancestor with 2n = 6, which likely had a chromosome set that was similar to that of *M*. *spirale* and *M*. *hystrix* (and *M*. *tuba*, and *M*. *finlandense*). Moreover, it seems possible that a tetraploidization or partial tetraploidization event took place early in karyotype evolution in the clade containing *M*. *lignano* and *Macrostomum* sp. 8, and that the additional chromosome material was later rearranged into the large chromosomes. However, in order to reconstruct karyotype evolution in this taxon, additional studies are clearly required to identify syntenic regions in the different studied species.

In many taxa, chromosome evolutionary breakpoint regions (EBRs) involved in chromosomal rearrangements are characterized by a high density of repetitive elements, structural variants, and/or segmental duplications [27, 28]. The formation of the *M*. *lignano* karyotype has probably passed through structural chromosome rearrangements involving such EBRs, which appear to be hotspots of evolutionary activity. However, we found no chromosomal regions that are strongly enriched with repeats. Neither DAPI and CMA staining, nor FISH with microdissected DNA probes have revealed any clear indication of such regions. Moreover, no interstitial telomeric sequences (ITSs) were revealed by our FISH experiments. Probably chromosome evolution in the *M*. *lignano* lineage, if via EBRs, has been accompanied by the elimination of regions enriched with repetitive DNA, including telomeric repeats.

We found clusters of ribosomal genes of different sizes in the chromosomes of *M*. *lignano*. Such polymorphism in sizes of rDNA clusters is common in other taxa, including some species of plants (e.g., garlic, *Allium subvillosum*), insects (e.g., cricket, *Anurogryllus* sp.), amphibians (e.g., salamander, *Ambystoma jeffersonianum*), and mammals (e.g., domestic pig, *Sus scrofa domesticus*) [29–32]. Thus, variation in the number and size of rDNA clusters, which in some cases can vary between homologous chromosomes, can not be considered as definite evidence for different composition of the chromosomes. The question of the evolutionary origin of the *M*. *lignano* karyotypes therefore remains open.

The most striking finding of our study, however, was that *M*. *lignano* showed unexpectedly high levels of intraspecific karyotype diversity. In animals, karyotype diversity in natural populations is mainly associated with Robertsonian translocations, B chromosomes, inversions of chromosome regions, and variation in the size of C-positive regions of chromosomes (i.e. regions containing constitutive heterochromatin blocks) derived from amplification of repetitive DNA [33, 34]. Also, whole-genome duplication (WGD) can be considered as a variant of karyotype diversity, and it often leads to viable polyploids and results in less pronounced effects on the phenotype and fitness than aneuploidy [35]. Moreover, numerous traces of multiple rounds of past WGD have been observed in the genomes of both plants and animals [36–38]. With the exception of WGDs, these types of karyotype reorganization do not often lead to changes in gene copy number or gene dosage imbalance [35, 39]. A few examples exist of karyotype diversity associated with gene dosage imbalance, for instance in the grasshopper, *Eyprepocnemis plorans*, which can include B chromosomes that can contain numerous repeats, i.e. retrotransposons and also B chromosomes containing histone or ribosomal genes [40, 41].

In the present study, karyotyping of individual specimens of *M*. *lignano* has revealed high levels of aneuploidy and in some cases other numerical and structural chromosome abnormalities among worms reared in the laboratory. Worms with tri- and tetrasomy of the large chromosome had a normal phenotype without evident morphological abnormalities and have been successfully maintained and cultured over extended periods of time. The same origin for the additional large chromosomes was indirectly confirmed by the results from the DAPI-banding (i.e. the same DAPI-positive patterns for normal and additional copies of the large chromosomes) and the crossing experiment (i.e. the additional copy of the large chromosome is usually normally inherited). Therefore, it is unlikely that the additional large chromosome(s) in *M*. *lignano* arose as the result of complex chromosomal rearrangements or chromosomal rearrangements involved massive DNA amplification.

In contrast to polyploidy described in many taxa, whole chromosome aneuploidy often leads to serious developmental deficiencies, diseases, and lethality [35, 42]. For example, studies of aneuploidy in different model species of plants (*Datura stramonium*, *Zea mays*), invertebrates (*Caenorhabditis elegans*, *Drosophila melanogaster*), and mammals (*Mus musculus*, *Homo sapiens*) have documented that aneuploidy has severe effects on development and growth [43–46]. Moreover, in mammals, autosomal aneuploidies are usually embryonic lethal [47]. In flatworms, cases of aneuploidy and different levels of polyploidy have previously been described in some fissiparous planarian species, including *Dugesia etrusca*, *Dugesia gonocephala*, *Dugesia benazii*, and *Polycelis nigra* [48–51]. For instance, after 10 years of maintenance of *D*. *etrusca* under laboratory conditions polyploid somatic cells and oocytes were revealed in two morphological races, namely *biadenodactyla* and *labronica* [48]. However, we observed many cases of trisomy, tetrasomy, and even monosomy for small and large chromosomes in laboratory-reared lines of *M*. *lignano* with no visible abnormalities in morphology and mostly normal fertility (unpublished data). This particularity of *M*. *lignano* makes this species uniquely suitable to study aneuploidy in animals.

The fact that we also found aneuploid worms in natural populations suggests that the discovered karyotype polymorphism is not solely the result of rearing worms in the laboratory. Given the very low frequency of these aneuploidies in natural populations, it is likely that laboratory conditions are more favorable for aneuploidy compared to natural populations. Several studies have shown that some model organisms (e.g. *Saccharomyces cerevisiae*) can display various levels of aneuploidy in laboratory conditions [52–54], and some authors have suggested that the duplication of chromosomes may actually offer an advantage by raising the dosage of a large set of genes, some of which may be beneficial under particular selective pressures encountered in the laboratory [53]. Unknown factors of the cultivation methods or the specific genetic background of the animals used to generate the inbred lines and outbred cultures could potentially have increased the frequency of aneuploid worms in the laboratory. Indeed, the aneuploidy levels tended to be higher in the investigated inbred lines compared to the outbred cultures. Unfortunately, only two inbred lines (DV1 and HUB1) were screened in this study, and the HUB1 line is a transgenic line derived from DV1, so these cannot be considered independent inbred lines. In the LS1 and IBK2 outbred cultures the frequency of abnormal karyotypes containing 9 or 10 chromosomes was rather low and no abnormal karyotypes were found in the LS2 culture. But aneuploid karyotypes were revealed in about half of studied worms in the LS3 culture, which stems from a different source population.

The high frequency of large chromosome tri- and tetrasomy in the studied inbred lines suggests that inbred animals with this type of aneuploidy may have a selective advantage compared to the animals showing the 'normal' 2n = 8 karyotype, at least under the specific laboratory conditions. This suggestion is in a good agreement with the lower than expected number of individuals with the 2n = 8 karyotype, both among individuals in the inbred DV1 population (Table 3) and among offspring of the controlled crosses (Table 4), suggesting a process that selects against inbred individuals with the 'normal' karyotype. Given that these lines are strongly inbred [14], one possibility is that this is caused by a form of maintained polymorphism, which protects the animals from becoming homozygous at specific loci, which would happen more often when they carry only two copies of that chromosome. The observed lower reproduction of these inbred lines compared to the outbred lines (L. Schärer, pers. obs.) may thus in part be due to considerable numbers of selective deaths among individuals that newly become homozygous for the large chromosome (or a specific chromosome region on that chromosome).

The results of the chromosome analysis of the IBK2 culture require special consideration. Different variants of numerical and structural chromosome rearrangements were revealed in this culture by single worm karyotyping, including additional submetacentric chromosomes, chromosomes of unknown origin and different numerical combinations of large and small chromosomes. The IBK2 culture was established from specimens of a natural population (Table 1) and at the time of analysis was heavily infected with single-celled eukaryotes (likely thraustochytrids; L. Schärer, pers. obs.) [55]. While we have no direct evidence of induction of structural chromosome rearrangements by thraustochytrids, we cannot exclude that this parasitic infestation could be a factor for the occurrence of structural chromosome abnormalities in IBK2 worms. Moreover, such an influence could either be a direct one caused by cytotoxic chemicals released by the single-cell eukaryotes, or it could act via a stress-induced misregulation of defense mechanisms against transposable elements, which occur in great abundance and diversity in the *M*. *lignano* genome [12].

Taking into account various chromosome abnormalities revealed in laboratory lines and cultures, and natural populations of *M*. *lignano*, we suspect that meiosis in this species is characterized by a high frequency of mistakes leading to numerical and structural chromosome abnormalities. Unfortunately, there currently is no published data available on meiosis in this species. The study of meiosis in another free-living flatworm, *D*. *etrusca*, revealed aneuploidy and polyploidy transmitted through the female germ line, whereas worms produced only normal spermatozoa [56]. Studies of another free-living flatworm, *Mesostoma ehrenbergi*, revealed particular features of meiosis (extensive chromosome oscillations, the absence of a metaphase plate, distance segregation of univalents, and a precocious ‘pre-anaphase’ cleavage furrow [57, 58]), which potentially can lead to formation of aneuploid gametes. While the features of meiosis in *M*. *lignano* could in theory be similar to those described in *M*. *ehrenbergi* and could thus potentially help to understand the chromosome aneuploidy revealed in this study, this does not appear very likely given that these species are only very distantly related, with *M*. *lignano* belonging to the early branching clade Macrostomorpha [5] and *M*. *ehrenbergi* belonging to the fairly derived clade Dalytyphloplanida, a group of the rhabdocoel flatworms [59].

Duplicated genes often undergo subfunctionalization and neofunctionalization. In these cases a polyploidization event, even if only partial, might fuel long-term diversification of the karyotype and evolutionary plasticity [60, 61]. Detailed analyses of a range of studied animal genomes have revealed a plethora of paralogous genes and paralogous chromosomal regions, pointing to an important role for ancient WGD or partial genome duplication in genome evolution [62, 63]. Investigations of the initial stages of genome evolution through WGD or partial chromosomal duplication are thus very important for the establishment of a proper evolutionary understanding of these evolutionary phenomena. It appears likely that in natural populations of *M*. *lignano* we observe the initial stages of the introduction of additional genetic material into the genome, providing raw material for evolutionary innovation. The first assembly of the *M*. *lignano* genome (DV1 line) indeed suggests a high frequency of genomic duplications [12], which compare well with the karyotypic data obtained in the present study. The *M*. *lignano* genome may have undergone a recent tetraploidization event and our karyotypic studies may provide clues about the recent evolutionary history of *M*. *lignano* chromosome rearrangements after this event. For instance, it could be that the large chromosome in the *M*. *lignano* karyotype has arisen as a result of chromosomal fusions of the additional chromosomal material resulting from the genome duplication. Therefore, the 2n = 8 worms could represent hidden tetraploids, and the 2n = 9 and 2n = 10 worms might thus essentially have pentaploid and hexaploid genomes, respectively. Such a scenario of a straight ploidy series could explain why the individuals of *M*. *lignano* having one or two additional copies of the large chromosome do not show severe abnormalities and can even produce the offspring. The verification of this hypothesis requires the comparative sequencing of the genomes of both normal and aneuploid animals. The discovery of karyotype diversity combined with normal phenotype in specimens of *M*. *lignano* characterized with tri- and tetrasomy of the large chromosome opens new avenues in the study of this interesting model organism. For example, comparative genome sequencing of specimens with normal karyotypes and different variants of aneuploid forms (tri- and tetrasomy of large chromosomes) will allow identifying the specific DNA content of the large chromosome.

Specimens with tetrasomy of the large chromosome might be considered as animals with a duplication of a large part of the basic genome. The changes of duplicated genes could lead to new features in the animals and as consequence to the appearance of new species with a more complex genome. From this point of view, the finding of *Macrostomum* sp. 8 is very promising. It is possible that *Macrostomum* sp. 8 derived from an ancestor similar to *M*. *lignano* through tetrasomy of the large chromosome. The large chromosome of *M*. *lignano* differs from both large chromosomes of *Macrostomum* sp. 8 in the location of the 28S rDNA cluster. This difference should not be considered a strong argument against the suggestion of a common ancestry of the large chromosomes of the two species, as many closely related species having the same karyotypes can differ in the sizes and locations of rDNA clusters [64]. Additional molecular markers for the different regions of these large chromosomes should be used to clarify this question. Comparisons of the karyology of *M*. *lignano*, *Macrostomum* sp. 8, and other species in the genus *Macrostomum* thus promise to provide interesting insights into karyotype and chromosome evolution.

## References

[1] Ladurner P, Schärer L, Salvenmoser W, Rieger RM. A new model organism among the lower Bilateria and the use of digital microscopy in taxonomy of meiobenthic Platyhelminthes: *Macrostomum lignano*, n. sp. (Rhabditophora, Macrostomorpha). J Zool Syst Evol Res. 2005;43(2):114–126.

[2] Mouton S, Willems M, Braeckman BP, Egger B, Ladurner P, Schärer L, et al The free-living flatworm *Macrostomum lignano*: a new model organism for ageing research. Exp Gerontol. 2009;44:243–249. 10.1016/j.exger.2008.11.007 19111920

[3] Simanov D, Mellaart-Stravert I, Sormacheva I, Berezikov E. The flatworm *Macrostomum lignano* is a powerful model organism for ion channel and stem cell research. Stem Cells Int. 2012; 167265. 10.1155/2012/167265 PMC3447372 23024658

[4] Schärer L, Ladurner P. Phenotypically plastic adjustment of sex allocation in a simultaneous hermaphrodite. P Roy Soc Lond B Bio. 2003;270:935–941. 10.1098/rspb.2002.2323 PMC1691333 12803908

[5] Janssen T, Vizoso DB, Schulte G, Littlewood DTJ, Waeschenbach A, Schärer L. The first multi-gene phylogeny of the Macrostomorpha sheds light on the evolution of sexual and asexual reproduction in basal Platyhelminthes. Mol Phylogenet Evol. 2015;92;82–107. 10.1016/j.ympev.2015.06.004 26093054

[6] Ramm SA, Schlatter A, Poirier M, Schärer L. Hypodermic self-insemination as a reproductive assurance strategy. P Roy Soc Lond B Bio. 2015;282:20150660. 10.1098/rspb.2015.0660 PMC4528547 26136446

[7] Ladurner P, Egger B, De Mulder K, Pfister D, Kuales G, Salvenmoser W, et al The stem cell system of the basal flatworm *Macrostomum lignano* In: Bosch TCG, editor. Stem Cells: from *Hydra* to Man. Berlin: Springer; 2008 p. 75–94.

[8] Wagner DE, Wang IE, Reddien PW. Clonogenic neoblasts are pluripotent adult stem cells that underlie planarian regeneration. Science. 2011;332:811–816. 10.1126/science.1203983 21566185 PMC3338249

[9] Egger B, Ladurner P, Nimeth K, Gschwentner R, Rieger R. The regeneration capacity of the flatworm *Macrostomum lignano*–on repeated regeneration, rejuvenation, and the minimal size needed for regeneration. Dev Genes Evol. 2006;216:565–577. 10.1007/s00427-006-0069-4 16604349 PMC2441584

[10] Pfister D, de Mulder K, Philipp I, Kuales G, Hrouda M, Eichberger P, et al The exceptional stem cell system of *Macrostomum lignano*: screening for gene expression and studying cell proliferation by hydroxyurea treatment and irradiation. Front Zool. 2007; 10.1186/1742-9994-4-9 17349046 PMC1828727

[11] Pfister D, de Mulder K, Hartenstein V, Kuales G, Borgonie G, Marx F, et al Flatworm stem cells and the germ line: developmental and evolutionary implications of macvasa expression in *Macrostomum lignano*. Dev Biol. 2008;319(1):146–159. 10.1016/j.ydbio.2008.02.045 18405892

[12] Wasik K, Gurtowski J, Zhou X, Ramos OM, Delás MJ, Battistoni G, et al Genome and transcriptome of the regeneration-complement flatworm, *Macrostomum lignano*. PNAS, 2015;112(40):12462–12467. 10.1073/pnas.1516718112 26392545 PMC4603488

[13] Schärer L, Littlewood DTJ, Waeschenbach A, Yoshida W, Vizoso DB. Mating behavior and the evolution of sperm design. PNAS. 2011;108(4):1490–1495. 10.1073/pnas.1013892108 21220334 PMC3029721

[14] Janicke T, Marie-Orleach L, De Mulder K, Berezikov E, Ladurner P, Vizoso DB, et al Sex allocation adjustment to local sperm competition in a simultaneous hermaphrodite. Evolution. 2013;67(11):3233–3242. 10.1111/evo.12189 24152005

[15] Demircan T. Advancing the flatworm Macrostomum lignano as a versatile model organism for stem cell research [dissertation]. Utrecht: Hubrecht Institute of the Royal Netherlands Academy of Arts and Sciences; 2013.

[16] Demircan T, Berezikov E. The hippo pathway regulates stem cells during homeostasis and regeneration of the flatworm *Macrostomum lignano*. Stem Cells Dev. 2013; 22:2174–2185. 10.1089/scd.2013.0006 23495768

[17] Marie-Orleach L, Janicke T, Vizoso D, Eichmann M, Schärer L. Fluorescent sperm in a transparent worm: validation of a GFP marker to study sexual selection. BMC Evol Biol. 2014; 10.1186/1471-2148-14-148 24980980 PMC4107727

[18] Egger B, Ishida S. Chromosome fission or duplication in *Macrostomum lignano* (Macrostomorpha, Plathelminthes)–remarks on chromosome numbers in ‘archoophoran turbellarians’. J Zool Syst Evol Res. 2005;43(2):127–132.

[19] Rieger R, Gehlen M, Hazprunar G, Homlund M, Legniti A, Salvenmoser W, et al Laboratory cultures of marine Macrostomida (Turbellaria). Forts Zool. 1988;36:525.

[20] Egger B, Gschwentner R, Hess MW, Nimeth KT, Adamski Z, Willems M, et al The caudal regeneration blastema is an accumulation of rapidly proliferating stem cells in the flatworm *Macrostomum lignano*. BMC Dev Biol. 2009;9:41 10.1186/1471-213X-9-41 19604404 PMC2717932

[21] Zadesenets KS, Karamysheva TV, Katokhin AV, Mordvinov VA, Rubtsov NB. Distribution of repetitive DNA sequences in chromosomes of five opisthorchid species (Trematoda, Opisthorchiidae). Parasitology Int. 2012;61(1):84–86. 10.1016/j.parint.2011.06.027 21791251

[22] Kim ES, Punina EO, Rodionov AV. Chromosome CPD (PI/DAPI)—and CMA-DAPI-banding patterns in *Allium cepa L*. Russ J Genet+. 2002;38(4):392–398. 12018166

[23] Yamamoto M, Takada N, Hirabayashi T, Kubo T, Tominaga S. Fluorescent staining analysis of chromosomes in pear (*Pyrus* spp.) J Japan Soc Hort Sci. 2010;79(1):23–26.

[24] Reeves A. MicroMeasure: a new computer program for the collection and analysis of cytogenetic data. Genome. 2001;44(3):439–443. 11444703

[25] Levan A. Nomenclature for centromeric position on chromosomes. Hereditas. 1964;52(2):201.

[26] Jido JW, Wells RA, Baldini A, Reeders ST. Improved telomere detection using a telomere repeat probe (TTAGGG)n generated by PCR. Nucleic Acids Res. 1991;19(17):4780 1891373 10.1093/nar/19.17.4780 PMC328734

[27] Rubtsov NB, Rubtsova NV, Anopriyenko OV, Karamysheva TV, Shevchenko AI, Mazurok NA, et al Reorganization of the X chromosome in voles of the genus *Microtus*. Cytogenet Genome Res. 2002;99:323–329. 12900582 10.1159/000071611

[28] Larkin DM, Pape G, Donthu R, Auvil L, Welge M, Lewin HA. Breakpoint regions and homologous synteny blocks in chromosomes have different evolutionary histories. Genome Res. 2009;19(5):770–777. 10.1101/gr.086546.108 19342477 PMC2675965

[29] Jamilena M, Ruiz-Rejón C, Ruiz-Rejón M. Variation in the heterochromatin and nucleolar organizing regions of *Allium subvillosum* L. (Liliaceae). Genome. 1990;33(6):779–784.

[30] Bi K, Bogart JP, Fu J. A populational survey of 45S rDNA polymorphism in the Jefferson salamander *Ambystoma jeffersonianum* revealed by fluorescence *in situ* hybridization (FISH). Current Zoology. 2009;55(2): 145–149.

[31] Schneider MC, Zacaro AA, Ferreira A, Cella DM. Karyotype plasticity in crickets: numerical, morphological, and nucleolar organizer region distribution pattern of *Anurogryllus* sp. J Insect Sci. 2010;10:87 10.1673/031.010.8701 20673072 PMC3383408

[32] Danielak-Czech B, Babicz M, Kozubska-Sobocinska A, Rejduch B. Size polymorphism survey of nucleolar organizer regions (NORs) in Hampshire boars. Annales Universitatis Mariae Curie-Skłodowska. Sectio EE: Zootechnica. 2013;31(4):8–13.

[33] Ruvinsky A, Graves JAM. Mammalian genomics Wallingford, Oxfordshire, UK; Cambridge, MA, USA, CABI Pub. 2005; 600 p.

[34] Graphodatsky AS, Trifonov VA, Stanyon R. The genome diversity and karyotype evolution of mammals. Molecular Cytogenetics. 2011;4:2 10.1186/1755-8166-4-22 21992653 PMC3204295

[35] Torres EM, Williams BR, Amon A. Aneuploidy: cells losing their balance. Genetics. 2008;179:737–746. 10.1534/genetics.108.090878 18558649 PMC2429870

[36] Storchova Z, Pellman D. From polyploidy to aneuploidy, genome instability and cancer. Nat Rev Mol Cell Bio. 2004;5:45–54. 14708009 10.1038/nrm1276

[37] Adams KL, Wendel JF. Polyploidy and genome evolution in plants. Curr Opin Plant Biol. 2005;8(2):135–141. 10.1016/j.pbi.2005.01.001 15752992

[38] Otto SP. The evolutionary consequences of polyploidy. Cell. 2007;131:452–462. 10.1016/j.cell.2007.10.022 17981114

[39] Hauffe H, Pialek J. Evolution of the chromosomal races of *Mus musculus domesticus* in the Rhaetian Alps: the roles of whole-arm reciprocal translocation and zonal raciation. Biol J Linn Soc. 1997;62(2):255–278.

[40] Marschner S, Meister A, Blatther FR, Houben A. Evolution and function of B chromosome 45S rDNA sequences in *Brachycome dichromosomatica*. Genome. 2007;50(7):638–644. 10.1139/g07-048 17893741

[41] Banaei-Moghaddam AM, Martis MM, Macas J, Gundlach H, Himmelbach A, Altschmied L, et al Genes on B chromosomes: old questions revisited with new tools. Biochimica et Biophysica Acta (BBA)–Gene Regulatory Mechanisms. 2015;1849(1):64–70. 25481283 10.1016/j.bbagrm.2014.11.007

[42] Niwa O, Tange Y, Kurabayashi A. Growth arrest and chromosome instability in aneuploid yeast. Yeast. 2006;23:937–950. 10.1002/yea.1411 17072887

[43] Blakeslee AF, Belling J, Farnham ME. Chromosomal duplication and Mendelian phenomena in *Datura* mutants. Science. 1920;52(1347): 388–390. 10.1126/science.52.1347.388 17829955

[44] Hernandez D, Fisher E. Mouse autosomal trisomy, two’s company, three’s a crowd. Trends Genet. 1999;15:241–247. 10354585 10.1016/s0168-9525(99)01743-6

[45] Altug-Teber O, Bonin M, Walter M, Mau-Holzmann UA, Dufke A, Stappert H, et al Specific transcriptional changes in human fetuses with autosomal trisomies. Cytogenet Genome Res. 2007;119(3–4):171–184. 10.1159/000112058 18253026

[46] Birchler JA, Veitia A. Gene balance hypothesis: connecting issues of dosage sensitivity across biological disciplines. PNAS. 2012;109(37): 14746–14753. 10.1073/pnas.1207726109 22908297 PMC3443177

[47] Luthardt FW, Keitges E. Chromosomal syndromes and genetic disease. eLS. 2001; 10.1038/npg.els.0001446

[48] Benazzi Lentati G. Gametogenesis and egg fertilization in Planarians. Int Rev Cytol. 1970;27: 101–179.

[49] Benazzi Lentati G, Deri P, Puccinelli I. Karyometric analysis on populations of *Dugesia benazii* (Turbellaria, Tricladid) evidencing a chromosome polymorphism. Atti Soc. Tosc Sci Nat, Mem, Serie B. 1987;94: 357–368.

[50] De Vries EJ. On the karyology of *Dugesia gonocephala* s.l. (Turbellaria, Tricladid) from Montpellier, France. Hydrobiologia. 1986;132:251–256.

[51] Beukeboom LW, Sharbel TF, Michiels NK. Reproductive modes, ploidy distribution, and supernumerary chromosome frequencies of the flatworm *Polycelis nigra* (Platyhelminthes: Tricladida). Hydrobiologia. 1998;383(1):277–285.

[52] Siegel JJ, Amon A. New insights into the troubles of aneuploidy. Annu Rev Cell Dev Biol. 2012;28:189–214. 10.1146/annurev-cellbio-101011-155807 22804579 PMC3919630

[53] Yona AH, Manor YS, Herbst RH, Romano GH, Mitchell A, Kupiec M, et al Chromosomal duplication is a transient evolutionary solution to stress. PNAS. 2012;109(51):21010–21015. 10.1073/pnas.1211150109 23197825 PMC3529009

[54] Chen G, Rubinstein B, Li R. Whole chromosome aneuploidy: big mutations drive adaptation by phenotypic leap. Bioessays. 2012;34(10): 893–900. 10.1002/bies.201200069 22926916 PMC3526072

[55] Schärer L, Vizoso DB, Rieger G, Peintner U. Thraustochytrids as novel parasitic protists of marine free-living flatworms: *Thraustochytrium caudivoru* sp. nov. parasitizes *Macrostomum lignano*. Marine Biology. 2007;152:1095–1104.

[56] Benazzi Lentati G. Sul determinismo e sulla ereditarietà della aneuploidia in *Dugesia etrusca benazzi* planaria a riproduzione anfigonica. Caryologia. 1957;10(2):352–387.

[57] Croft JA, Jones GH. Meiosis in *Mesostoma ehrenbergii ehrenbergii*. IV. Recombination nodules in spermatocytes and a test of the correspondence of late recombination nodules and chiasmata. Genetics. 1989;121(2): 255–262. 2731723 10.1093/genetics/121.2.255 PMC1203615

[58] Ferraro-Gideon J, Hoang C, Forer A. *Mesostoma ehrenbergii* spermatocytes—a unique and advantageous cell for studying meiosis. Cell Biol Int. 2013; 10.1002/cbin.10130 23686688

[59] Willems WR, Wallberg A, Jondelius U, Littlewood DTJ, Backeljau T, Schockaert ER, et al Filling a gap in the phylogeny of flatworms: relationships within the Rhabdocoela (Platyhelminthes), inferred from 18S ribosomal DNA sequences. Zool. Scr. 2006;35:1–17.

[60] Comai L. The advantages and disadvantages of being polyploid. Nat Rev Genet. 2005;6: 836–846. 10.1038/nrg1711 16304599

[61] McGrath CL, Lynch M. Evolutionary significance of whole-genome duplication In: Soltis PS, Soltis DE, editors. Polyploidy and genome evolution. Berlin Heidelberg: Springer-Verlag; 2012 p. 1–21.

[62] Pébusque MJ, Coulier F, Birnbaum D, Pontarotti P. Ancient large-scale genome duplication: phylogenetic and linkage analyses shed light on chordate genome evolution. Mol Biol Evol. 1998;15(9):1145–1159. 9729879 10.1093/oxfordjournals.molbev.a026022

[63] Jackson AP. Evolutionary consequences of a large duplication event in *Trypanosoma brucei*: chromosomes 4 and 8 are partial duplicons. BMC Genomics. 2007;8:432 10.1186/1471-2164-8-432 18036214 PMC2212663

[64] Panzera Y, Pita S, Ferreiro MJ, Ferrandis I, Lages C, Perez R, et al High dynamics of rDNA cluster location in kissing bug holocentric chromosomes (Triatominae, Heteroptera). Cytogenet Genome Res. 2012;138(1):56–67. 10.1159/000341888 22907389

